# Chronic Insomnia and Military Separation: A Retrospective Matched Cohort and Nested Case-Control Study of U.S. Active Component Service Members, 2014–2023

**Published:** 2026-08-20

**Authors:** Zachary J. Rupert, Shauna L. Stahlman, Michael T. Fan, Symone M. Baker Miller, Nicole M. Hsu

**Affiliations:** Uniformed Services University of the Health Sciences, Bethesda, MD: Lt Col Rupert; Epidemiology and Analysis Branch, Armed Forces Health Surveillance Division, Public Health Directorate, Defense Health Agency, Silver Spring, MD: Dr. Stahlman, LCDR Baker Miller, Dr. Fan; Immunization Healthcare Division, Public Health Directorate, Defense Health Agency, Falls Church, VA: Lt Col Hsu

## Abstract

This study characterized chronic insomnia disorder, utilizing data from the Defense Medical Surveillance System, to identify incident cases among U.S. active component service members from 2014 through 2023. A total of 126,733 service members met the case definition and were matched 1-to-1 with service members without chronic insomnia by sex, age, and time in service. Chronic insomnia was associated with increased probability of early separation from military service and frequently co-occurred with conditions that limit military readiness. Kaplan-Meier analysis showed diverging retention curves approximately 20 months following diagnosis, after which chronic insomnia cases had lower probability of continued active component service. A nested case-control analysis of 55,679 individuals with chronic insomnia found that early separation was associated with service branch, age, sex, race and ethnicity, marital status, rank, occupation, co-occurring diagnoses, and behavioral therapy or pharmacotherapy within 365 days of diagnosis. Behavioral therapy or pharmacotherapy within 365 days of diagnosis was more common among those who separated early. Continued attention to case surveillance, early identification, and timely evidence-based treatment may help mitigate the impacts of insomnia on service outcomes.

Chronic insomnia disorder is characterized by difficulty falling asleep, difficulty staying asleep, or early morning awakening, causing significant distress or daytime impairment, occurring 3 times or more per week for at least 3 months and not explained by another condition or inadequate sleep opportunity. ^1^ Chronic insomnia is associated with reduced resilience, impaired functioning, and a range of negative occupational outcomes. ^2 - 4^ As many as one half of U.S. adults experience symptoms of insomnia, and the estimated prevalence of chronic insomnia disorder ranges 5–23%. ^5 - 8^ Military service members are at increased risk due to occupational factors such as operational stress, irregular work schedules, and deployments. ^2 , 3^

A recent surveillance report identified over 148,000 active component service members (ACSMs) with incident chronic insomnia disorder from 2012 through 2021. ^3^ Observed rate differences suggested a disproportionate burden by branch of service, sex, age, and race and ethnicity, with higher rates among Army members, women, older service members, and non-Hispanic Black service members. ^3^

**What are the new findings?**

Service members with chronic insomnia face increased risk of early separation from service in the military. Early separation was more common among service members who had received behavioral therapy or pharmacotherapy within the first year after diagnosis. A high burden of co-occurring conditions was identified, including behavioral health disorders, traumatic brain injury, and chronic pain.

**What is the impact on readiness and force health protection?**

Chronic insomnia may serve as an early indicator of increased risk of separation from military service and is often concomitant with other conditions. Efforts to emphasize sleep health as a readiness factor, with a focus on improved preventive measures, in addition to early identification and timely use of evidence-based treatment, may help mitigate the impacts of insomnia on the health of the force.

Chronic insomnia may also affect military readiness because of its relationship with behavioral health disorders and neurological conditions, which are major contributors to disability evaluation and retention outcomes. ^9 , 10^ Sleep disorders frequently coexist with behavioral health disorders and neurological conditions; evidence suggests these relationships are often interrelated, with conditions mutually exacerbating each other. ^3 , 5 , 6 , 8^ Initial Physical Evaluation Board (PEB) evaluations serve as the proxy for retention medical standards, and data from fiscal year 2023 consistently emphasized high involvement of both behavioral health and neurological conditions. ^9 , 10^ Specifically, behavioral health conditions alone were involved in 35–47% of initial PEB evaluations among all service branches, while neurological conditions were involved in 20–26%. ^9 , 10^ Among those referred for disability evaluation, over 40% of unfitting diagnoses involved mental health or neurological disorders. ^10^ While insomnia was not listed as a primary condition, it is frequently comorbid with other mental health diagnoses and cited as a potential contributing factor to over-all impairment and unfit determinations. ^3 , 10^

Effective, evidence-based treatments for chronic insomnia are available. ^6 , 11^ Cognitive behavioral therapy for insomnia (CBT-I) is the primary intervention recommended in both military and civilian clinical practice guidelines (CPGs). ^5 , 6 , 11^ Short-term pharmacotherapy may be used selectively, but is not recommended as a primary or long-term strategy. ^6 , 11^

Although chronic insomnia is associated with impaired functioning and adverse occupational outcomes, less is known about its relationship with military-specific outcomes, including continued service and early separation. This report addresses 2 specific questions: 1) Do service members diagnosed with chronic insomnia have shorter lengths of active component service following diagnosis compared to matched peers without insomnia? and 2) Among those diagnosed with chronic insomnia, what demographic, medical, or treatment factors are associated with completing service versus separating early for medical or negative administrative reasons?

## Methods

### Data source and chronic insomnia case definition

The Defense Medical Surveillance System (DMSS) was utilized to identify insomnia diagnoses using International Classification of Diseases, 9th and 10th revisions (ICD-9 / ICD-10), with an incident case defined as having at least 2 inpatient or outpatient medical encounters in any diagnostic position, separated by 90–390 days. ^3 , 7^ The first qualifying encounter was used as the date of diagnosis. The surveillance period was January 1, 2014 through December 31, 2023 and included all individuals serving in the active component of the U.S. Army, Navy, Air Force, Space Force, or Marine Corps. Exclusion criteria included National Guard, reserve, and Coast Guard personnel, and individuals with an insomnia diagnosis prior to the study period. This study was conducted as part of the Armed Forces Health Surveillance Division’s routine public health surveillance mission.

### Matched cohort analysis

To assess the association between chronic insomnia and separation from service, ACSMs with chronic insomnia, who were required to be in military service at the time of the case diagnosis, were matched 1-to-1 to an unexposed matched cohort of ACSMs without any insomnia diagnoses during their military careers. Additional matching criteria included sex, age (±365 days), and cumulative time in service (±365 days) as of the case’s insomnia diagnosis. Branch of service was not included as a matching variable to avoid overly restrictive matching and potential loss of eligible matched pairs; sensitivity testing confirmed that matching by service branch did not significantly alter the survival curve. Members of the unexposed matched cohort were assigned the same index date as their corresponding chronic insomnia cases.

The primary outcome was separation from active component military service for any reason. The Kaplan–Meier survival method was used to estimate the probability of remaining in active service over time among chronic insomnia cases and the unexposed matched cohort. The log-rank test was used to evaluate whether there was a statistically significant difference between the survival curves. Demographic and occupational variables were assessed as of the index date.

### Nested case-control analysis

A nested case-control analysis was conducted among ACSMs with chronic insomnia who separated from active component service during the study period. The objective was to evaluate whether treatment factors, common mental health and neurological comorbidities, or demographics were associated with completing a term of service versus separating early for medical or negative administrative reasons.

Eligible individuals were ACSMs who met the case definition for chronic insomnia during the surveillance period. Individuals had to remain in continuous active service for at least 1 year following diagnosis. This criterion ensured enough follow-up time to evaluate medical care and co-occurring diagnoses that occurred prior to separation. Separation records were reviewed to classify outcomes based on Interservice Separation Codes (ISCs), which are assigned at the time of discharge and documented on the official record of separation from military service, the Certificate of Release or Discharge from Active Duty, or DD214.

In the nested case-control analysis, cases were individuals with chronic insomnia who experienced “early separation,” defined as separation due to medical disability or negative administrative reasons prior to completion of service obligation. The comparison group consisted of ‘termcompletion controls’, defined as individuals with chronic insomnia who completed their obligated terms of service. The ISCs used to classify early separation and term completion are listed in **Table 1** .

**TABLE 1.** Inter-Service Codes Associated with Early Separation and Term Completion, Active Component, U.S. Armed Forces

Code	Description
Early Separation	
1010	Condition existing prior to service
1011	Disability, severance pay
1012	Permanent disability retirement
1013	Temporary disability retirement
1014	Disability, no condition existing prior to service, no severance pay
1017	Failure to meet weight or body fat standards
1060	Character or behavior disorder
1062	Enuresis
1064	Alcoholism
1065	Discreditable incidents, civilian or military
1066	Shirking
1067	Drugs
1068	Financial Irresponsibility
1069	Lack of dependent support
1071	Civil court conviction
1072	Security
1073	Court-martial
1074	Fraudulent entry
1075	AWOL or desertion
1079	Juvenile offender
1080	Misconduct, reason unknown
1083	Pattern of minor disciplinary infractions
1084	Commission of a serious offense
1085	Failure to meet minimum qualifications for retention
1086	Unsatisfactory performance (former Expeditious Discharge Program)
1087	Entry level performance and conduct (former Trainee Discharge Program)
1088	Unsatisfactory performance of Ready Reserve obligation
1098	Breach of contract
1101	Dropped from strength, desertion
1102	Dropped from strength, imprisonment
2010	Condition existing prior to service
2011	Disability, severance pay
2012	Permanent disability retirement
2013	Temporary disability retirement
2017	Failure to meet weight or body fat standards
2060	Character or behavior disorder
2061	Motivational problems (apathy)
2063	Failure of course of instruction
2064	Alcoholism
2065	Discreditable incidents, civilian or military
2067	Drugs
2068	Financial Irresponsibility
2071	Civil court conviction
2072	Security
2073	Court martial
2074	Fraudulent entry
2078	Good of the service (discharge in lieu of court martial)
2083	Pattern of minor disciplinary infractions
2084	Commission of a serious offense
2085	Failure to meet minimum retention requirements
2102	Dropped from strength, imprisonment
Term Completion	
1001	Expiration of term of service
1050	Retirement, 20-30 years of service
1051	Retirement, 30+ years of service
1052	Retirement, other
2001	Expiration of term of service
2050	Retirement, 20-30 years of service
2051	Retirement, 30+ years of service
2052	Retirement, other
2053	Retirement, failure of selection for promotion

Abbreviation: AWOL, absent without leave.

### Treatment exposure and co-occurring diagnoses

Treatment exposure was defined as receipt of CPG-concordant insomnia care within 365 days of diagnosis. Behavioral therapy (BT) was identified using outpatient medical encounters that included both an insomnia diagnosis and qualifying Current Procedural Terminology (CPT) codes for psychotherapy, as described by Hsu et al. ^3^ Because administrative data do not confirm the specific psychotherapy content delivered, BT exposure was categorized as: no therapy, brief therapy (defined as 1–3 sessions), and full CBT-I (defined as >=4 sessions). Pharmacotherapy (PT) was defined by the presence of a prescription for CPG-recommended medications. ^6^ Timing of treatment initiation (within 90 days vs. later or no treatment) was also assessed.

Covariates included demographic and military characteristics, as well as co-occurring diagnoses for anxiety, depression, post-traumatic stress disorder (PTSD), bipolar disorder, substance use disorders, adjustment disorder, traumatic brain injury (TBI), and chronic pain, captured from inpatient or outpatient records within ±365 days of the insomnia diagnosis. Mental health conditions were identified using ICD-9 / ICD-10 code sets consistent with the methodology employed in the *MSMR* update on mental health disorders; TBI was identified using codes specified by the Armed Forces Health Surveillance Division surveillance case definition. ^12 , 13^ Chronic pain was identified using ICD-9 code 338.2 and ICD-10 codes G89.29 and G89.4.

### Statistical analysis

Descriptive statistics were used to compare treatment patterns and comorbidities for types of separation. A multi-variable logistic regression model was used to obtain adjusted odds ratios (aORs) for early separation. The final model included branch of service, sex, age, race and ethnicity, marital status, rank, occupation, alcohol or substance use disorder, TBI, chronic pain, anxiety, depressive disorder, adjustment disorder, PTSD, bipolar disorder, and receipt of any CPG-concordant therapy within 365 days.

## Results

A total of 126,751 ACSMs met the surveillance case definition for chronic insomnia. A total of 18 chronic insomnia cases were excluded because no matched service member was identified within 1 year of cumulative time in service. Total cases by year, as well as baseline demographic and military characteristics, are shown in **Table 2** . The largest demographic groups were Army soldiers, men, non-Hispanic White individuals, senior enlisted service members, those never deployed, those with less than 5 years of service at diagnosis, and those in communications or intelligence and repair or engineering occupations.

**TABLE 2.** Service Members Diagnosed with Chronic Insomnia and Matched Unexposed Cohort Used in Time-to-Separation Analysis, Active Component, U.S. Armed Forces, 2014–2023

Characteristics	Chronic Insomnia		Unexposed Matched Cohort		Chi-square *p* -value ^a^
	No.	%	No.	%	
Total	126,733	100	126,733	100	
Year of matching, diagnosis					1.0000
2014	11,215	8.8	11,215	8.8	
2015	12,902	10.2	12,902	10.2	
2016	14,044	11.1	14,044	11.1	
2017	12,587	9.9	12,587	9.9	
2018	11,137	8.8	11,137	8.8	
2019	11,844	9.3	11,844	9.3	
2020	11,192	8.8	11,192	8.8	
2021	13,741	10.8	13,741	10.8	
2022	14,163	11.2	14,163	11.2	
2023	13,908	11.0	13,908	11.0	
Branch of service					<0.0001
Army	71,497	56.4	39,480	31.2	
Navy	20,675	16.3	35,580	28.1	
Air Force	23,011	18.2	33,121	26.1	
Marine Corps	11,550	9.1	18,552	14.6	
Sex					1.0000
Male	97,077	76.6	97,077	76.6	
Female	29,656	23.4	29,656	23.4	
Race and ethnicity					<0.0001
White, non-Hispanic	60,288	47.6	72,903	57.5	
Black, non-Hispanic	32,615	25.7	19,775	15.6	
Hispanic	20,015	15.8	18,956	15.0	
Other	11,505	9.1	12,919	10.2	
Unknown	2,310	1.8	2,180	1.7	
Marital status					<0.0001
Single	38,439	30.3	46,473	36.7	
Married	77,348	61.0	73,089	57.7	
Other	10,925	8.6	7,060	5.6	
Unknown	21	0.0	111	0.1	
Age, *y*					<0.0001
<20	3,778	3.0	6,355	5.0	
20–24	30,233	23.9	30,713	24.2	
25–29	27,714	21.9	26,612	21.0	
30–34	20,274	16.0	19,647	15.5	
35–39	23,083	18.2	21,772	17.2	
40+	21,651	17.1	21,634	17.1	
Rank					<0.0001
Junior enlisted (E1–E4)	46,172	36.4	46,064	36.3	
Senior enlisted (E5–E9)	65,188	51.4	56,567	44.6	
Junior officer (O1–O3)	5,914	4.7	10,209	8.1	
Senior officer (O4–O10)	6,368	5.0	11,763	9.3	
Warrant officer	3,091	2.4	2,130	1.7	
Military occupation					<0.0001
Infantry, artillery, armor, combat engineering	17,697	14.0	15,718	12.4	
Motor transport	4,375	3.5	3,431	2.7	
Pilot, air crew	1,560	1.2	5,514	4.4	
Repair, engineering	32,105	25.3	38,094	30.1	
Communications, intelligence	34,512	27.2	28,036	22.1	
Health care	15,360	12.1	11,411	9.0	
Other	21,124	16.7	24,529	19.4	
Deployment prior to matching					<0.0001
Never deployed	61,542	48.6	63,790	50.3	
1 deployment	23,792	18.8	23,610	18.6	
2+ deployments	41,399	32.7	39,333	31.0	
Time in service at matching, *y*					<0.0001
<5	46,949	37.0	49,962	39.4	
5<=10	26,852	21.2	25,262	19.9	
10<=18	24,893	19.6	24,753	19.5	
18+	28,039	22.1	26,756	21.1	

Abbreviations: No., number; *y* , years.

a Significant *p* -values (<0.0001) for matched variables are an artifact of displaying continuous ±365-day matches in categorical groups.

### Time-to-separation analysis

Matched survival analysis was conducted using 126,733 chronic insomnia cases and an equal number of matched service members in the matched unexposed cohort. Time to separation was tracked beginning at the index insomnia diagnosis for cases and the corresponding match date for matched service members without chronic insomnia.

The Kaplan-Meier survival curves **(Figure)** display descriptive estimates of the probability of retention over time. The curves differed significantly between chronic insomnia cases and the matched unexposed cohort ( *p* <0.01). The curves cross between 19 and 20 months, after which service members with chronic insomnia had a lower estimated probability of continued active duty service. By 24 months after index diagnosis, 45.6% of chronic insomnia cases remained on active duty compared to 48.5% of the matched unexposed cohort. At 24, 48, and 100 months after the index date, retention rates were 45.6%, 26.5%, and 12.3%, respectively, among chronic insomnia cases, compared with 48.5%, 32.8%, and 19.1% in the matched unexposed cohort.

**FIGURE:**
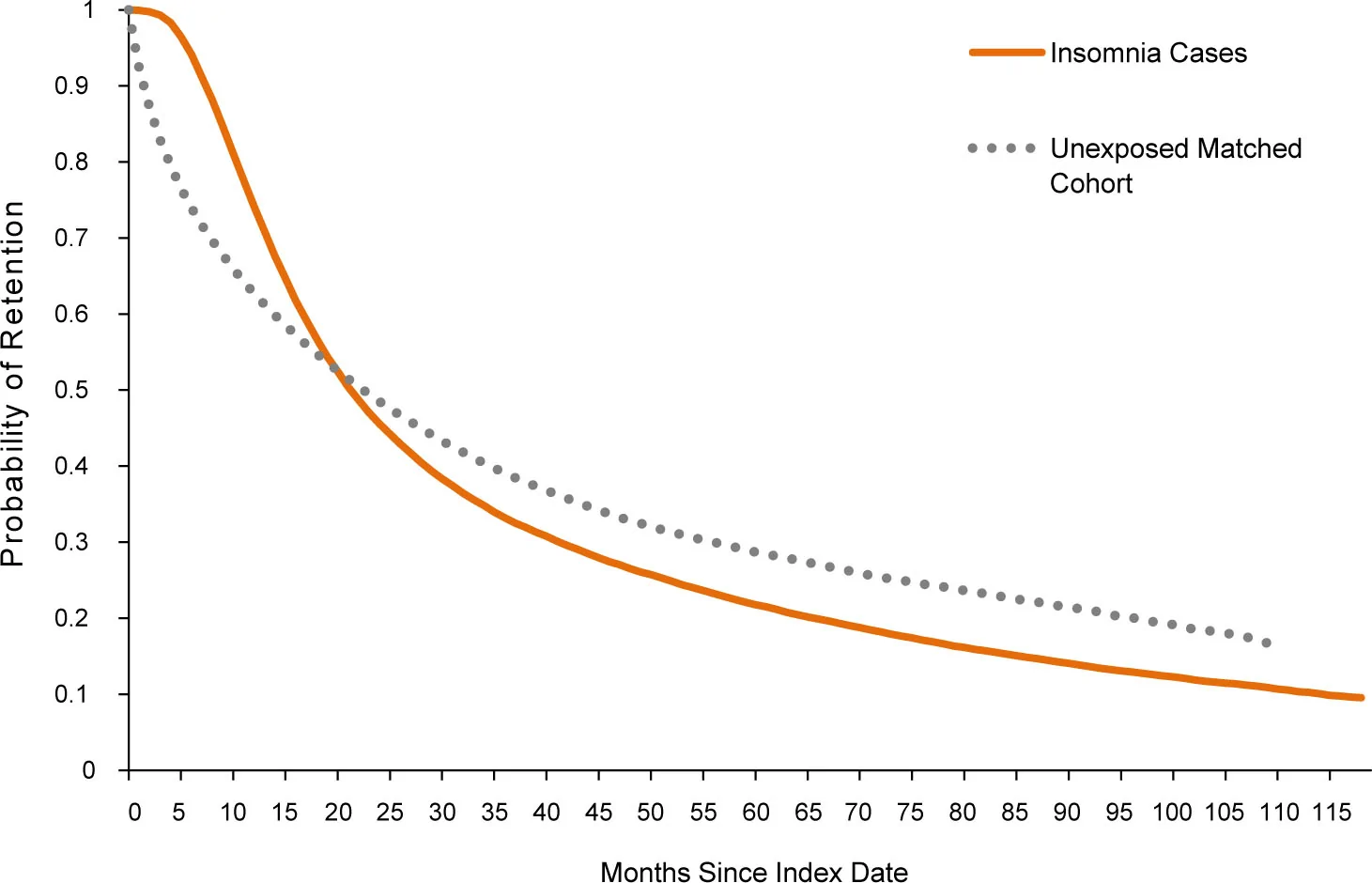
Probability of Retention of Service Members with Chronic Insomnia, Active Component, U.S. Armed Forces, 2014–2023

### Demographic, medical and treatment factors association with separation

The nested case-control analysis included 55,679 service members with chronic insomnia who separated from active duty during the surveillance period. Of these, 29,282 experienced early separation and 26,397 were term-completion controls **(Table 3)** .

**TABLE 3.** Demographic and Medical Factors, Diagnoses of Chronic Insomnia, Early Separation Versus Term Completion Controls, Active Component, U.S. Armed Forces, 2014–2023

Characteristics	Early Separation Cases		Term-Completion Controls		Odds Ratio	95% CI LL	95% CI UL	*p* -value
	No.	%	No.	%				
Total	29,282	100	26,397	100				
Branch of service								
Army	20,678	70.6	12,202	46.2	Reference			
Navy	3,238	11.1	4,816	18.2	0.40	0.38	0.42	<0.0001
Air Force	3,225	11.0	6,106	23.1	0.31	0.30	0.33	<0.0001
Marine Corps	2,141	7.3	3,273	12.4	0.39	0.36	0.41	<0.0001
Sex								
Male	21,907	74.8	21,322	80.8	Reference			
Female	7,375	25.2	5,075	19.2	1.41	1.36	1.47	<0.0001
Race and ethnicity								
White, non-Hispanic	13,745	46.9	12,789	48.4	Reference			
Black, non-Hispanic	8,344	28.5	6,758	25.6	1.15	1.10	1.20	<0.0001
Hispanic	4,500	15.4	3,869	14.7	1.08	1.03	1.14	0.0017
Other	2,174	7.4	2,301	8.7	0.88	0.83	0.94	<0.0001
Unknown	519	1.8	680	2.6	0.71	0.63	0.80	<0.0001
Marital status								
Single	9,182	31.4	5,094	19.3	Reference			
Married	17,744	60.6	18,437	69.8	0.53	0.51	0.56	<0.0001
Other	2,352	8.0	2,863	10.8	0.46	0.43	0.49	<0.0001
Unknown	4	0.0	3	0.0	0.74	0.17	3.31	0.6931
Age, *y*								
<20	1,171	4.0	266	1.0	Reference			
20–24	8,104	27.7	3,283	12.4	0.56	0.49	0.64	<0.0001
25–29	7,376	25.2	2,887	10.9	0.58	0.51	0.67	<0.0001
30–34	5,606	19.1	2,533	9.6	0.50	0.44	0.58	<0.0001
35–39	4,018	13.7	7,871	29.8	0.12	0.10	0.13	<0.0001
40+	3,007	10.3	9,557	36.2	0.07	0.06	0.08	<0.0001
Rank								
Junior enlisted (E1–E4)	13,026	44.5	4,593	17.4	Reference			
Senior enlisted (E5–E9)	13,793	47.1	17,066	64.7	0.29	0.27	0.30	<0.0001
Junior officer (O1–O3)	1,359	4.6	922	3.5	0.52	0.48	0.57	<0.0001
Senior officer (O4–O10)	571	2.0	2,781	10.5	0.07	0.07	0.08	<0.0001
Warrant officer	533	1.8	1,035	3.9	0.18	0.16	0.20	<0.0001
Military occupation								
Infantry, artillery, armor, combat engineering	4,732	16.2	3,263	12.4	Reference			
Motor transport	1,296	4.4	740	2.8	1.21	1.09	1.34	0.0002
Pilot, air crew	211	0.7	459	1.7	0.32	0.27	0.38	<0.0001
Repair, engineering	6,989	23.9	6,660	25.2	0.72	0.68	0.77	<0.0001
Communications, intelligence	7,765	26.5	7,819	29.6	0.69	0.65	0.72	<0.0001
Health care	3,521	12.0	3,236	12.3	0.75	0.70	0.80	<0.0001
Other	4,768	16.3	4,220	16.0	0.78	0.73	0.83	<0.0001
Deployment prior to incident diagnosis								
Never deployed	13,878	47.4	6,332	24.0	Reference			
1 deployment	6,518	22.3	5,264	19.9	0.57	0.54	0.59	<0.0001
2 + deployments	8,886	30.3	14,801	56.1	0.27	0.26	0.29	<0.0001
Time in service at incident diagnosis, *y*								
<5	8,182	27.9	2,259	8.6	Reference			
5<=10	9,214	31.5	4,102	15.5	0.62	0.58	0.66	<0.0001
10<=18	7,117	24.3	1,711	6.5	1.15	1.07	1.23	0.0001
18+	4,769	16.3	18,325	69.4	0.07	0.07	0.08	<0.0001
Co-occurring diagnoses within ± 365 days of incident diagnosis								
Alcohol, substance use disorder	5,648	19.3	2,252	8.5	2.56	2.43	2.70	<0.0001
Anxiety disorder	16,303	55.7	11,121	42.1	1.73	1.67	1.78	<0.0001
Depressive disorder	15,738	53.7	8,487	32.2	2.45	2.37	2.54	<0.0001
Adjustment disorder	19,364	66.1	11,982	45.4	2.35	2.27	2.43	<0.0001
PTSD	10,549	36.0	6,445	24.4	1.74	1.68	1.81	<0.0001
Bipolar disorder	993	3.4	200	0.8	4.60	3.95	5.36	<0.0001
TBI	5,874	20.1	3,009	11.4	1.95	1.86	2.05	<0.0001
Chronic pain	11,512	39.3	6,251	23.7	2.09	2.01	2.17	<0.0001
None	2,256	7.7	6,144	23.3				

Abbreviations: No., number; CI, confidence interval; LL, lower limit; UL, upper limit; *y* , years; PTSD, post-traumatic stress disorder; TBI, traumatic brain injury.

The Army constituted the largest share of the study population and had the highest proportion of early separations, evidencing differences in separation outcomes among the service branches. Separation outcomes by demographic subgroup showed variations in the proportions of early separation versus term completion in relation to sex, race and ethnicity, marital status, age, rank, occupation, deployment history, and time in service. Service members with 1 or more prior deployments had lower unadjusted odds of early separation than those who had never deployed. Service members with 18 or more years of service at diagnosis had a substantially higher proportion of term completion. Within occupational categories, motor transport had higher unadjusted odds of early separation compared with the infantry, artillery, armor, combat engineering category, while pilot and air crew, repair and engineering, communications and intelligence, health care, and other occupations had lower unadjusted odds.

**Table 3** also shows early separation and term completion outcomes by co-occurring diagnoses. The diagnoses were not mutually exclusive. As a descriptive summary of the co-occurring diagnosis burden, individuals were classified as having at least 1 listed co-occurring diagnosis if they had any of the selected diagnoses included in **Table 3** within ±365 days of a chronic insomnia diagnosis. Of the 55,679 individuals included in the nested case-control study, only 8,400 (15.1%) had no listed co-occurring diagnoses.

**Table 4** lists insomnia treatments received by individuals within 365 days of initial diagnosis, highlighting differences between the early separation and term completion groups, including receipt of behavioral therapy (categorized as ‘no therapy’, ‘brief therapy with 1–3 encounters’, or ‘full CBT-I with 4+ encounters’) and receipt of any CPG-concordant pharmaco-therapy. A greater proportion of early separation cases received behavioral therapy or CPG-concordant pharmacotherapy within 365 days of diagnosis.

**TABLE 4.** Treatment Factors, Chronic Insomnia Diagnoses, Early Separation Versus Term Completion Controls, Active Component, U.S. Armed Forces, 2014–2023

Characteristics	Early Separation Cases		Term-Completion Controls		Odds Ratio	95% CI LL	95% CI UL	*p* -value
	No.	%	No.	%				
Total	29,282	100	26,397	100				
Behavioral therapy within 365 days on, after diagnosis								
Yes	18,041	61.6	12,169	46.1	1.88	1.81	1.94	<0.0001
No	11,241	38.4	14,228	53.9	Reference			
CPG-recommended pharmacotherapy within 365 days on, after diagnosis								
Yes	7,602	26.0	5,455	20.7	1.35	1.29	1.40	<0.0001
No	21,680	74.0	20,942	79.3	Reference			
Behavioral therapy or CPG-recommended pharmacotherapy within 365 days on, after diagnosis								
Yes	20,626	70.4	14,832	56.2	1.86	1.79	1.92	<0.0001
No	8,656	29.6	11,565	43.8	Reference			
Behavioral therapy sessions within 365 days on, after diagnosis, *n*								
1–3	9,749	33.3	7,136	27.0	1.73	1.66	1.80	<0.0001
4+	8,292	28.3	5,033	19.1	2.09	2.00	2.18	<0.0001
None	11,241	38.4	14,228	53.9	Reference			
Behavioral therapy within 90 days on, after diagnosis								
Yes	13,269	45.3	8,837	33.5	1.65	1.59	1.70	<0.0001
No	16,013	54.7	17,560	66.5	Reference			
CPG-recommended pharmacotherapy within 90 days on, after diagnosis								
Yes	3,874	13.2	3,100	11.7	1.15	1.09	1.21	<0.0001
No	25,408	86.8	23,297	88.3	Reference			
Behavioral therapy or CPG-recommended pharmacotherapy within 90 days on, after diagnosis								
Yes	15,258	52.1	10,831	41.0	1.56	1.51	1.62	<0.0001
No	14,024	47.9	15,566	59.0	Reference			
Behavioral therapy sessions within 90 days on, after diagnosis, *n*								
1–3	10,204	34.8	6,978	26.4	1.60	1.55	1.66	<0.0001
4+	3,065	10.5	1,859	7.0	1.81	1.70	1.92	<0.0001
None	16,013	54.7	17,560	66.5	Reference			

Abbreviations: No., number; CI, confidence interval; LL, lower limit; UL, upper limit; CPG, clinical practice guideline; *n* , number.

**Table 5** details the aORs for early separation. Compared with the Army, odds of early separation were significantly lower for Navy (aOR 0.37), Air Force (aOR 0.32), and Marine Corps (aOR 0.26) personnel. Female sex was associated with higher adjusted odds of early separation compared with male sex (aOR 1.26). Older age groups had lower adjusted odds of early separation compared with service members younger than age 20 years. Adjusted odds of early separation were higher among junior personnel than among senior enlisted personnel, senior officers, and warrant officers. Among the occupational categories, motor transport was associated with higher adjusted odds of early separation compared with the infantry, artillery, armor, combat engineering category (aOR 1.16), while communications and intelligence (aOR 0.89) and health care (aOR 0.89) had lower adjusted odds. Receipt of behavioral therapy or pharmacotherapy within 365 days of diagnosis was associated with higher adjusted odds of early separation (aOR 1.12). All listed co-occurring diagnoses were associated with increased odds of early separation.

**TABLE 5.** Adjusted Odds Ratios for Early Separation, Chronic Insomnia Diagnoses, Active Component, U.S. Armed Forces, 2014–2023

Characteristics	Adjusted Odds Ratio	95% CI LL	95% CI UL
Sex			
Male	Reference		
Female	1.26	1.19	1.32
Branch of service			
Army	Reference		
Navy	0.37	0.35	0.40
Air Force	0.32	0.30	0.34
Marine Corps	0.26	0.24	0.28
Age, *y*			
<20	Reference		
20–24	0.53	0.46	0.61
25–29	0.59	0.50	0.69
30–34	0.51	0.43	0.60
35–39	0.10	0.09	0.12
40+	0.07	0.05	0.08
Race and ethnicity			
White, non-Hispanic	Reference		
Black, non-Hispanic	1.08	1.03	1.14
Hispanic	1.01	0.95	1.07
Other	0.94	0.87	1.02
Unknown	1.12	0.97	1.28
Rank			
Junior enlisted (E1–E4)	Reference		
Senior enlisted (E5–E9)	0.59	0.55	0.63
Junior officer (O1–O3)	1.05	0.93	1.17
Senior officer (O4–O10)	0.37	0.33	0.42
Warrant officer	0.48	0.41	0.55
Military occupation			
Infantry, artillery, armor, combat engineering	Reference		
Motor transport	1.16	1.02	1.30
Pilot, air crew	1.03	0.84	1.28
Repair, engineering	1.02	0.95	1.09
Communications, intelligence	0.89	0.83	0.96
Health care	0.89	0.82	0.97
Other	1.08	1.00	1.17
Marital status			
Single	Reference		
Married	1.23	1.16	1.31
Other	1.06	0.97	1.15
Unknown	0.98	0.18	5.31
Co-occurring diagnoses within ± 365 days of chronic insomnia diagnosis			
No listed diagnosis	Reference		
Alcohol, substance use disorder	1.45	1.36	1.54
Anxiety disorder	1.17	1.12	1.22
Depressive disorder	1.67	1.59	1.74
Adjustment disorder	1.30	1.25	1.36
PTSD	1.66	1.58	1.75
Bipolar disorder	2.52	2.12	3.00
TBI	1.67	1.58	1.77
Chronic pain	2.06	1.97	2.16
Behavioral therapy or CPG-concordant pharmacotherapy within 365 days of chronic insomnia diagnosis			
No	Reference		
Yes	1.12	1.07	1.17

Abbreviations: CI, confidence interval; LL, lower limit; UL, upper limit; *y* , years; PTSD, post-traumatic stress disorder; TBI, traumatic brain injury.

## Discussion

Service members diagnosed with chronic insomnia demonstrated a reduced probability of continued active component service after 20 months compared with the matched unexposed cohort, with this difference sustained through the remainder of the surveillance period. The survival curve is likely influenced by the case definition, which required 2 insomnia-related encounters 90–390 days apart. This introduced a survivorship period of 3–13 months, during which, unlike individuals in the matched unexposed cohort, cases had to remain in service to be included. As a result, retention initially appeared higher among cases, but once that period ended, the curves converged and then crossed, indicating an overall higher probability of separation among those with chronic insomnia.

The nested case-control analysis high-lights several key patterns. Consistent with prior surveillance findings showing service-specific differences in chronic insomnia burden, Army members accounted for the largest number of chronic insomnia cases. ^3^ In this study, service members in the Navy, Air Force, and Marine Corps had lower adjusted odds of early separation compared with Army members. The reasons for these service-specific differences could not be determined from this analysis.

In the nested case-control analysis, early separation among ACSMs with chronic insomnia was associated with younger age, female sex, non-Hispanic Black race or ethnicity, current marriage, and motor transport occupation. Older age groups had lower adjusted odds of early separation compared with service members younger than 20 years, and senior enlisted personnel, senior officers, and warrant officers had lower adjusted odds compared with junior enlisted personnel. The associations with younger age and lower rank are expected within the context of this analysis, as these individuals are inherently less likely to reach retirement eligibility. Observed differences in chronic insomnia burden and associated separation risk by sex, race and ethnicity, and marital status are consistent, however, with findings from longitudinal military cohorts, such as the Millennium Cohort Study, and military surveillance reports. ^2 , 4^ These differences may reflect underlying social or structural factors, including unequal access to support resources, potential bias in administrative separation processes, or differences in exposure to occupational stressors. ^3 , 6 , 7^ Prior studies have identified differences by sex and race/ethnicity in chronic insomnia burden among service members and sociodemographic variation in insomnia-related care among veterans, supporting the need for further study of potential disparities in military populations. ^3 , 7^ These findings emphasize the need for further investigation into potential disparities in separation outcomes among demographic subgroups.

It is difficult to isolate a direct relationship between chronic insomnia and separation from service, in part, due to the high prevalence of co-occurring conditions. In this study, nearly 85% of individuals with chronic insomnia had at least 1 co-occurring diagnosis selected for analysis: anxiety, depression, PTSD, bipolar disorder, substance use disorders, adjustment dis-order, TBI, or chronic pain. The relationships between chronic insomnia and those co-occurring diagnoses are often inter-related, with insomnia potentially both a cause and a consequence. ^5 , 6^ Furthermore, these highly comorbid conditions, or the treatments used for their management (e.g., antipsychotics, benzodiazepines, hypnotics, intensive therapy), may render a service member non-deployable or unfit for duty, thereby mandating a Medical Evaluation Board (MEB) and subsequent PEB determination. ^6 , 9 , 10^ This required medical review directly factors into the odds ratios for early separation, as the PEB outcome serves as a proxy for service retention medical standards. ^9 , 10^ As such, insomnia may serve as a marker of underlying risk rather than a primary driver of separation. Future research should aim to link clinical diagnoses, including symptom severity, to documented reasons for separation.

Lastly, receipt of behavioral therapy or CPG-concordant pharmacotherapy within 365 days of chronic insomnia diagnosis was associated with higher adjusted odds of early separation (aOR 1.12). This should not be interpreted as evidence that treatment increased separation risk. Rather, treatment may have served as a proxy for greater symptom severity, persistence, functional impairment, or clinical recognition. Administrative data could not capture treatment fidelity, completion, symptom response, or duty-related functional outcomes. Prior research among active duty personnel suggests that CBT-I can improve insomnia symptoms, although clinically meaningful improvement may occur in only a subset of treated service members. ^14^ Future studies should evaluate whether timely, complete, and high-fidelity insomnia treatment improves functional outcomes and reduces early separation risk.

This study has several important limitations. It lacked clinical information on insomnia severity, symptom burden, functional impairment, and treatment response, which limits interpretation of clinical impact and its association with early separation. Specific medical conditions responsible for separation were also not available, meaning that some separations may have been driven by conditions unrelated to insomnia. Additionally, this analysis did not distinguish between medical and negative administrative separations, which likely involve different contributing factors. The case definition required 2 insomnia diagnoses 90–390 days apart, potentially excluding milder or short-term cases and skewing the sample toward more severe, persistent, or treatment-resistant insomnia. The reliance on administrative data from DMSS may not adequately capture self-managed cases and may reflect potential variability in clinical coding practices. Behavioral therapy exposure was also identified using diagnosis and CPT codes, which did not confirm whether CBT-I-specific components were delivered, whether treatment was completed, or whether symptoms improved. Similarly, ISCs are applied administratively and may not fully capture the complexity of discharge circumstances; their use may vary among service branches or over time, introducing potential bias into comparisons between early separation and term completion.

Despite these limitations, the findings offer important insights into the relationship between chronic insomnia and service outcomes. Chronic insomnia was associated with an increased probability of early separation and frequently co-occurred with behavioral health and neurological conditions. Although it may not be the primary cause of separation, insomnia may serve as a marker of broader risk, particularly when present with other comorbidities. Efforts to improve early identification, intervention, and adherence to clinical practice guidelines may help mitigate the impacts of insomnia on force health. Future studies and surveillance efforts would benefit from incorporating broader case definitions, assessing clinical severity, evaluating the impact of evidence-based insomnia therapies on separation outcomes, and prospectively linking clinical diagnoses to specific reasons for separation from military service. Enhanced surveillance and greater policy attention to sleep health may support force readiness and long-term retention.
